# Rab11-mediated post-Golgi transport of the sialyltransferase ST3GAL4 suggests a new mechanism for regulating glycosylation

**DOI:** 10.1016/j.jbc.2021.100354

**Published:** 2021-01-30

**Authors:** Masato Kitano, Yasuhiko Kizuka, Tomoaki Sobajima, Miyako Nakano, Kazuki Nakajima, Ryo Misaki, Saki Itoyama, Yoichiro Harada, Akihiro Harada, Eiji Miyoshi, Naoyuki Taniguchi

**Affiliations:** 1 Department of Glyco-Oncology and Medical Biochemistry, Osaka International Cancer Institute, Otemae, Chuo-ku, Osaka, Japan; 2 Department of Molecular Biochemistry and Clinical Investigation, Graduate School of Medicine, Osaka University, Osaka, Japan; 3 Center for Highly Advanced Integration of Nano and Life Sciences (G-CHAIN), Gifu University, Gifu, Japan; 4 Institute for Glyco-core Research (iGCORE), Gifu University, Gifu, Japan; 5 Graduate School of Integrated Sciences for Life, Hiroshima University, Hiroshima, Japan; 6 Fujita Health University, Aichi, Japan; 7 International Center for Biotechnology, Osaka University, Osaka, Japan; 8 Department of Cell Biology, Graduate School of Medicine, Osaka University, Osaka, Japan

**Keywords:** Rab11, sialyltransferase, glycosylation, ST3GAL4, α2,3-sialylation, N-linked glycosylation, membrane traffic, posttranslational modification, glycosyltransferase, glycan, B4GALNT2, beta-1,4-*N*-acetylgalactosaminyltransferase 2, B4GALT1, beta-1,4-galactosyltransferase 1, CHX, cycloheximide, COG, conserved oligomeric Golgi, ConA, concanavalin A lectin, DKD cells, HeLa cells in which both Rab11a and Rab11b are stably knocked down, EIC, extracted ion chromatogram, Endo H, endoglycosidase H, ER, endoplasmic reticulum, FUT8, fucosyltransferase 8, GlcAT-P, glucuronyltransferase-P, IKO, intestine-specific Rab11a knockout, L4-PHA, *Phaseolus vulgaris* Leucoagglutinin lectin, LC-ESI-MS, liquid chromatography–electrospray ionization–mass spectrometry, MAM, *Maackia amurensis* lectin, ManNAz, *N*-azidoacetylmannosamine, NeuAc, CMP-*N*-acetylneuraminic acid, PGC, post-Golgi compartment, PhoSL, *Pholiota squarrosa* lectin, PM, plasma membrane, PNGase F, peptide *N*-glycosidase F, PVDF, poly(vinylidene fluoride), RCA120, Ricinus Communis Agglutinin 120, SiaNAz, *N*-azidoacetyl sialic acid, sLe^X^, sialyl-Lewis^X^, SSA, *Sambucus sieboldiana* lectin, ST3GAL4, CMP-*N*-acetylneuraminate-beta-galactosamide-alpha-2,3-sialyltransferase 4, SDS-PAGE, SDS-polyacrylamide gel electrophoresis, ST6GAL1, Beta-galactoside alpha-2,6-sialyltransferase 1, ST8SIA1, Alpha-*N*-acetylneuraminide alpha-2,8-sialyltransferase, TBS, tris-buffered saline, TGN, trans-Golgi network, vWF, von Willebrand factor

## Abstract

Glycosylation, the most common posttranslational modification of proteins, is a stepwise process that relies on tight regulation of subcellular glycosyltransferase location to control the addition of each monosaccharide. Glycosyltransferases primarily reside and function in the endoplasmic reticulum (ER) and the Golgi apparatus; whether and how they traffic beyond the Golgi, how this trafficking is controlled, and how it impacts glycosylation remain unclear. Our previous work identified a connection between *N*-glycosylation and Rab11, a key player in the post-Golgi transport that connects recycling endosomes and other compartments. To learn more about the specific role of Rab11, we knocked down Rab11 in HeLa cells. Our findings indicate that Rab11 knockdown results in a dramatic enhancement in the sialylation of *N*-glycans. Structural analyses of glycans using lectins and LC-MS revealed that α2,3-sialylation is selectively enhanced, suggesting that an α2,3-sialyltransferase that catalyzes the sialyation of glycoproteins is activated or upregulated as the result of Rab11 knockdown. ST3GAL4 is the major α2,3-sialyltransferase that acts on *N*-glycans; we demonstrated that the localization of ST3GAL4, but not the levels of its mRNA, protein, or donor substrate, was altered by Rab11 depletion. In knockdown cells, ST3GAL4 is densely distributed in the trans-Golgi network, compared with the wider distribution in the Golgi and in other peripheral puncta in control cells, whereas the α2,6-sialyltransferase ST6GAL1 is predominantly localized to the Golgi regardless of Rab11 knockdown. This indicates that Rab11 may negatively regulate α2,3-sialylation by transporting ST3GAL4 to post-Golgi compartments (PGCs), which is a novel mechanism of glycosyltransferase regulation.

Glycosylation is one of the most common posttranslational modifications in eukaryotes and has a major role in regulating the functions of glycoproteins and cells, including folding, activity, cell adhesion, and communication (1). Glycan structures are diverse and dynamically change to enable complex multicellular systems such as immunity, the nervous system, and development (2, 3, 4). In addition, undesirable changes in glycan structures cause various diseases, including cancer and diabetes (5, 6, 7). Therefore, for a further understanding of the physiological and pathological roles of glycans, elucidating how glycan structures are altered is a pivotal issue.

Among various types of glycosylation, *N*-glycosylation is highly conserved in mammals (8). In the endoplasmic reticulum (ER), the common form of oligosaccharide is attached to an asparagine residue in the consensus sequence (Asn-X-Ser/Thr, X is except for Pro) (9, 10). During the transport of proteins from the ER to the Golgi apparatus, the attached *N*-glycans are processed and modified by the stepwise action of various glycosyltransferases (11). To date, approximately 180 human glycosyltransferases have been identified, and their expression levels impact the biosynthesis of protein-, cell-type-, and disease-specific glycans (12). In particular, the actions of glycosyltransferases localized in the Golgi are responsible for the structural diversity of *N*-glycans, and the expression level of the responsible glycosyltransferase is considered to be a primary factor for the biosynthesis of a certain glycan.

In addition to the expression level, the localization of glycosyltransferases is also a major factor for glycan biosynthesis. In the biosynthetic pathways of glycans, Golgi-localized glycosyltransferases often share an acceptor glycan and can compete with each other (13, 14). To the contrary, some glycosyltransferases form heterocomplexes in order to cooperate with each other (15, 16, 17). Therefore, each glycosyltransferase should be trafficked to its own appropriate place to maintain the glycan profiles in cells (18, 19). Indeed, the abnormal transport of glycosyltransferases significantly impacts glycan biosynthesis and causes the expression of aberrant glycans. For example, the deletion of Golgi scaffold proteins, Giantin or GM130, leads to the mislocalization of glycosyltransferases and impaired glycosylation (20). In addition, defects in the conserved oligomeric Golgi (COG) complex comprised of COG proteins and their interacting partners (Rab GTPases, golgins, and SNAREs) also lead to aberrant glycosylation probably due to the impaired transport of glycosyltransferases (21, 22). Despite the importance of the appropriate trafficking of glycosyltransferases for glycan expression, the mechanisms responsible for their regulation remain largely unclear. It is particularly noteworthy that little is known concerning whether and how glycosyltransferases are trafficked to the post-Golgi compartments (PGCs), including early/late/recycling endosomes and the plasma membrane (PM).

In our previous report, to our surprise, we found that the *N*-glycosylation of several glycoproteins is altered in mutant mice lacking Rab11a (23) that is involved in vesicular trafficking *via* recycling endosomes (24). In mammals, the Rab GTPase family is composed of at least 60 members, and they function to regulate intracellular membrane transport (25). Although Rab proteins that are involved in trafficking between the ER and the Golgi are likely to be committed to the transport of glycosyltransferases (26), the involvement of Rab11 proteins (Rab11a and Rab11b) in glycosylation has not been elucidated. Rab11 is frequently used as a marker of recycling endosomes and functions in intracellular recycling pathways, cholesterol transport, and autophagosome formation (27, 28, 29, 30). Although it has been reported that the pathological roles of Rab11 proteins are involved in Alzheimer’s disease, Huntington’s disease, and cancer (31), details of the *in vivo* physiological functions of Rab11 proteins remain poorly understood. Since Rab11a systemic knockout mice are embryonically lethal (32), we generated intestine-specific Rab11a knockout (IKO) mice and used them in an attempt to elucidate the physiological roles of Rab11a in the gut (23). In addition to the impaired trafficking of apical proteins to the apical membrane in the IKO mice, we discovered that the mobility of several glycoproteins in SDS-polyacrylamide gel electrophoresis (SDS-PAGE) gel was commonly shifted up in the mutant mice. Furthermore, the mobility shifts were canceled when the *N*-glycans were removed by treatment with peptide *N*-glycosidase F (PNGase F), strongly suggesting that abnormal *N*-glycosylation is caused by a Rab11a deficiency. However, the issue of how Rab11 is involved in the *N*-glycosylation pathway is not completely clear.

In this study, to reveal the mechanisms by which Rab11 regulates *N*-glycosylation, we investigated the detailed changes in *N*-glycans and glycosyltransferases in Rab11-knockdown HeLa cells. Using lectin blotting and mass spectrometry (MS) analyses, we found that the extent of sialylation, particularly α2,3-sialylation, is strikingly increased in Rab11-knockdown cells, which was likely caused by the accumulation of the α2,3-sialyltransferase ST3GAL4 in the Golgi. Our findings suggest the existence of a pathway in which this glycosyltransferase is trafficked to PGCs, highlighting the roles of Rab11 proteins as novel regulators of *N*-glycosylation.

## Results

### Aberrant N-glycosylation in Rab11a and Rab11b double-knockdown HeLa cells

**Figure 1:**
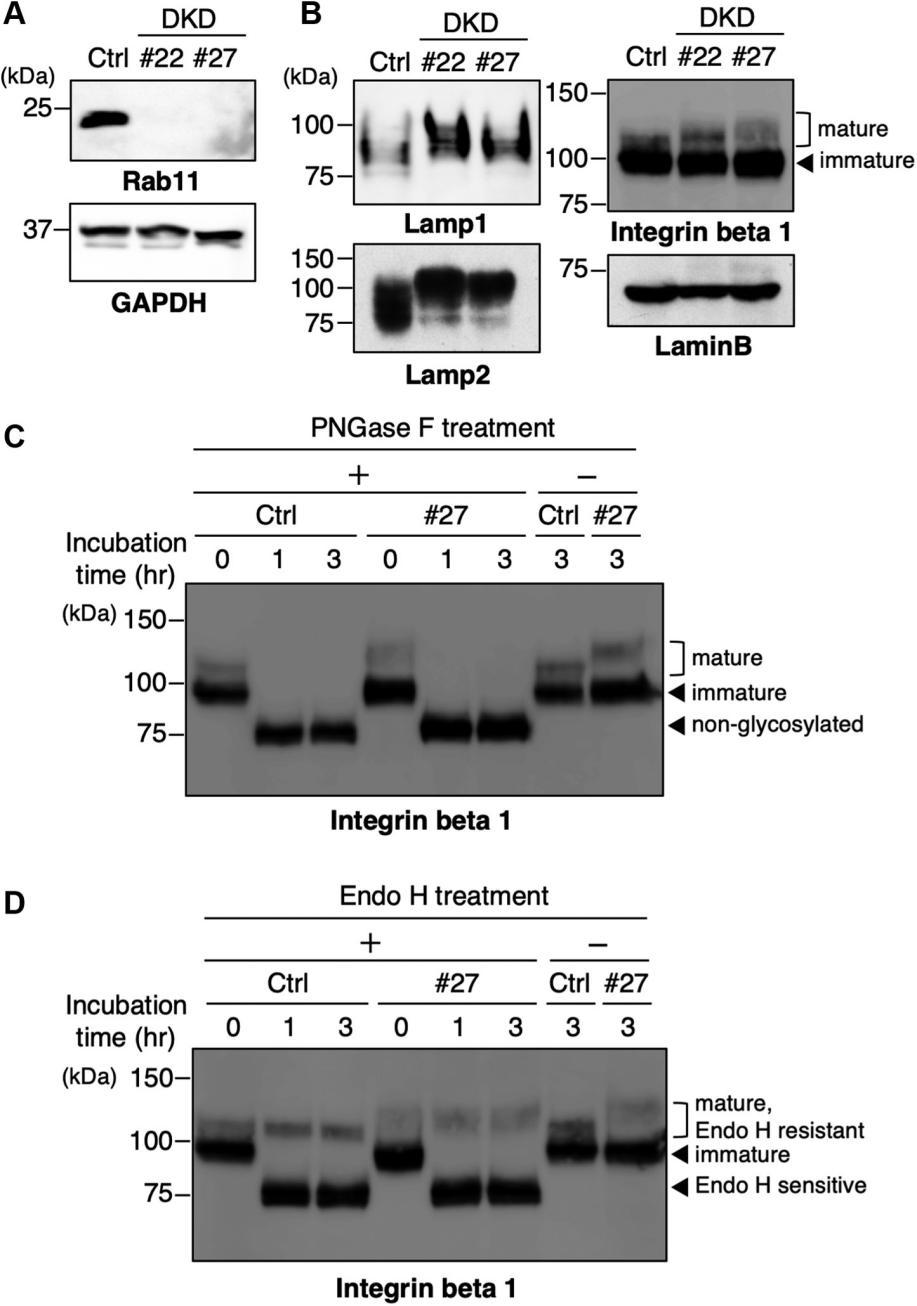
**Abnormal *N*-glycosylation in Rab11a and Rab11b double-knockdown HeLa cells**. *A*, the depletion efficiency of Rab11 proteins in two HeLa cell clones stably expressing shRNAs targeting Rab11a and Rab11b (DKD cells) (#22 and #27) was examined by western blotting with anti-Rab11 antibody that reacts with both Rab11 isoforms. *B*, proteins in control and DKD cells were analyzed by western blotting for several glycoproteins (Lamp1, Lamp2, integrin beta 1) and Lamin B (loading control). *C* and *D*, proteins from control and DKD cells were treated with PNGase F (*C*) or Endo H (*D*) for 0, 1, or 3 h, and then subjected to western blotting with an anti-integrin beta 1 antibody.

Our previous study using IKO mice strongly suggested that *N*-glycosylation is dysregulated by a Rab11 deficiency (23). To elucidate the roles of Rab11 proteins in *N*-glycosylation, we stably knocked down both Rab11a and Rab11b in HeLa cells (double knockdown (DKD) cells) (28). The levels of Rab11a and Rab11b in two representative clones of DKD cells (#22 and #27) were analyzed by western blotting with an antibody that reacts with both of the Rab11 isoforms (Fig. 1*A*). Compared with control cells, the expression levels of the Rab11 isoforms in both DKD clones were essentially negligible, confirming that both Rab11 isoforms were successfully silenced in these cells. To examine the issue of whether the abnormal *N*-glycosylation found in Rab11-deficient mice was recapitulated in DKD cells, we examined the mobility of heavily *N*-glycosylated proteins such as Lamp1, Lamp2, and integrin beta 1 by western blotting, which have over 10 *N*-glycosylation sites (33, 34). We found that the mobility of these proteins, but not that of the non-*N*-glycosylated protein Lamin B, was markedly slower in DKD cells compared with control cells (Fig. 1*B*), suggesting that *N*-glycosylation is disturbed in various glycoproteins in Rab11 DKD cells. To confirm that the mobility shifts were due to abnormal *N*-glycosylation, proteins from control and DKD cells were treated with peptide *N*-glycosidase F (PNGase F) to remove all *N*-glycans and analyzed by western blotting. After the PNGase F treatment, the mobility of integrin beta 1 from control and DKD cells converged, demonstrating that the slower mobility of glycoproteins in the DKD cells was due to altered *N*-glycosylation (Fig. 1*C*). It should also be noted that integrin beta 1 showed two bands in western blotting, and both forms converged after the PNGase F treatment (Fig. 1*C*). This indicates that these two bands are differentially glycosylated forms of integrin beta1, which is consistent with previous reports showing that the upper band represents a mature form localized to the cell surface and the lower band represents a premature form localized to the ER (35). Treatment with endoglycosidase H (Endo H), which cleaves only oligomannose and hybrid types of *N*-glycans but not complex type *N*-glycans, resulted in the downshift of only the lower band (Fig. 1*D*), further confirming that the mature and the immature forms are modified with complex and oligomannose or hybrid *N*-glycans, respectively. The mobility shift by the Rab11 knockdown was only observed for the mature form of integrin beta1 (Fig. 1, *B* and *D*), suggesting that Rab11a and Rab11b regulate steps at the late stage of *N*-glycan biosynthesis in the Golgi apparatus rather than the early stage in the ER.

### Sialylation is enhanced in DKD cells

**Figure 2:**
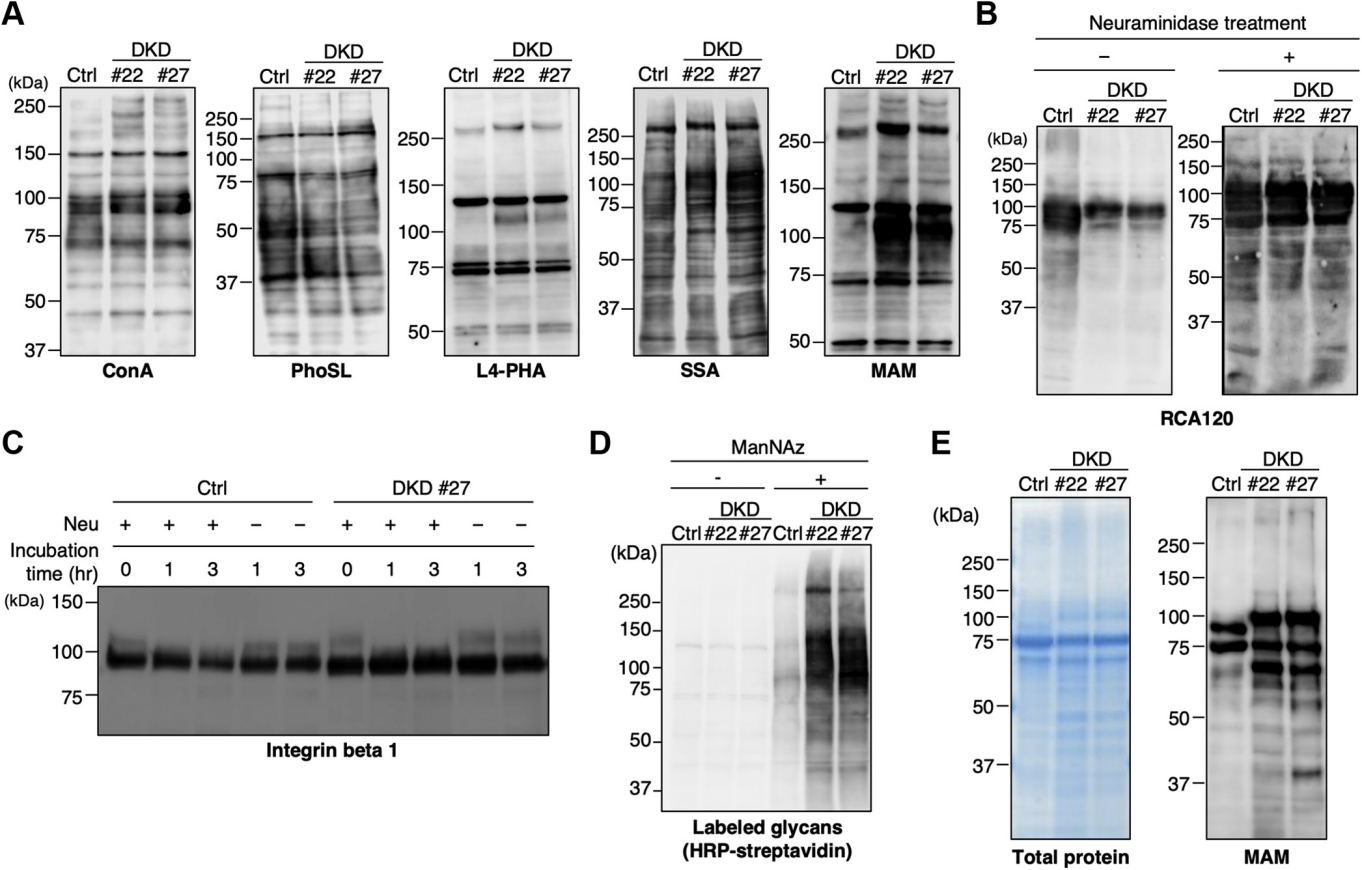
**Sialylation is enhanced in DKD cells.***A*, proteins from control and DKD cells were subjected to lectin blotting with ConA, PhoSL, L4-PHA, SSA, and MAM lectins. *B*, proteins from control and DKD cells were subjected to SDS-PAGE and transferred to membranes. After treatment with or without neuraminidase, the membranes were blotted with an RCA120 lectin. *C*, proteins from control and DKD cells were treated with neuraminidase for 0, 1, or 3 h, and then analyzed by western blotting with an anti-integrin beta 1 antibody. *D*, control and DKD cells were supplied with or without ManNAz. Proteins in the cell lysates were biotinylated by click reaction and subjected to SDS-PAGE and detection with HRP-conjugated streptavidin. *E*, extracellular proteins in the culture medium of control and DKD cells were subjected to SDS-PAGE. GelCode Blue staining (left) or blotting with MAM lectins (right) are shown.

To investigate how *N*-glycan structures are altered in DKD cells, we conducted lectin blotting with various lectins with different binding preferences (ConA, oligomannose glycans; PhoSL, core fucose; L4-PHA, β1,6-GlcNAc; SSA, α2,6Sia; MAM, α2,3Sia) (Fig. 2*A*). We observed slight or moderate increases in the reactivity of some proteins with ConA and L4-PHA in the DKD samples compared with control cells, and we observed only a few changes in the reactivity with PhoSL in DKD cells (Fig. 2*A*). More striking increases in reactivity with Sia-recognizing lectins, particularly MAM, were observed in the DKD cells, suggesting that the degree of sialylation is enhanced in DKD cells. To confirm this, we performed lectin blotting with β1,4Gal-recognizing RCA120, since the terminal capping of β1,4Gal with sialic acid blocks the binding of RCA120. Consistent with the increases in reactivity with Sia-recognizing lectins, we found a substantial reduction in RCA120 reactivity in DKD cells (Fig. 2*B*, left). A neuraminidase treatment canceled this reduction (Fig. 2*B*, right), further confirming that terminal sialylation is enhanced in DKD cells. The mobility shift of the upper band of integrin beta 1 in DKD cells was also canceled by a neuraminidase treatment (compare the second and seventh lanes, the third and eighth lanes in Fig. 2*C*), indicating that the decreased mobility of glycoproteins in DKD cells is due to enhanced sialylation. To further confirm that sialylation was potentiated in DKD cells, we visualized the level of sialylation in cells by means of a click chemistry approach (36). Control and DKD cells were incubated with equivalent amounts of *N*-azidoacetylmannosamine (ManNAz), which is converted to CMP-*N*-azidoacetyl sialic acid (SiaNAz) and transferred to glycoconjugates by sialyltransferases in cells. The detection of SiaNAz-labeled glycans by click chemistry showed that the level of labeled glycans in DKD cells had dramatically increased in comparison with control cells (Fig. 2*D*), further showing that sialylation is enhanced in DKD cells. We also found that proteins in culture media of DKD cells also displayed increased reactivity with the MAM lectin (Fig. 2*E*), excluding the possibility that the increased reactivity of DKD cells with the MAM lectin is caused by an impaired secretion of sialylated proteins. These collective data demonstrate that the knockdown of Rab11 enhances sialylation in the Golgi apparatus.

### MS glycan analysis revealed an increase in α2,3-sialylation in DKD cells

**Figure 3:**
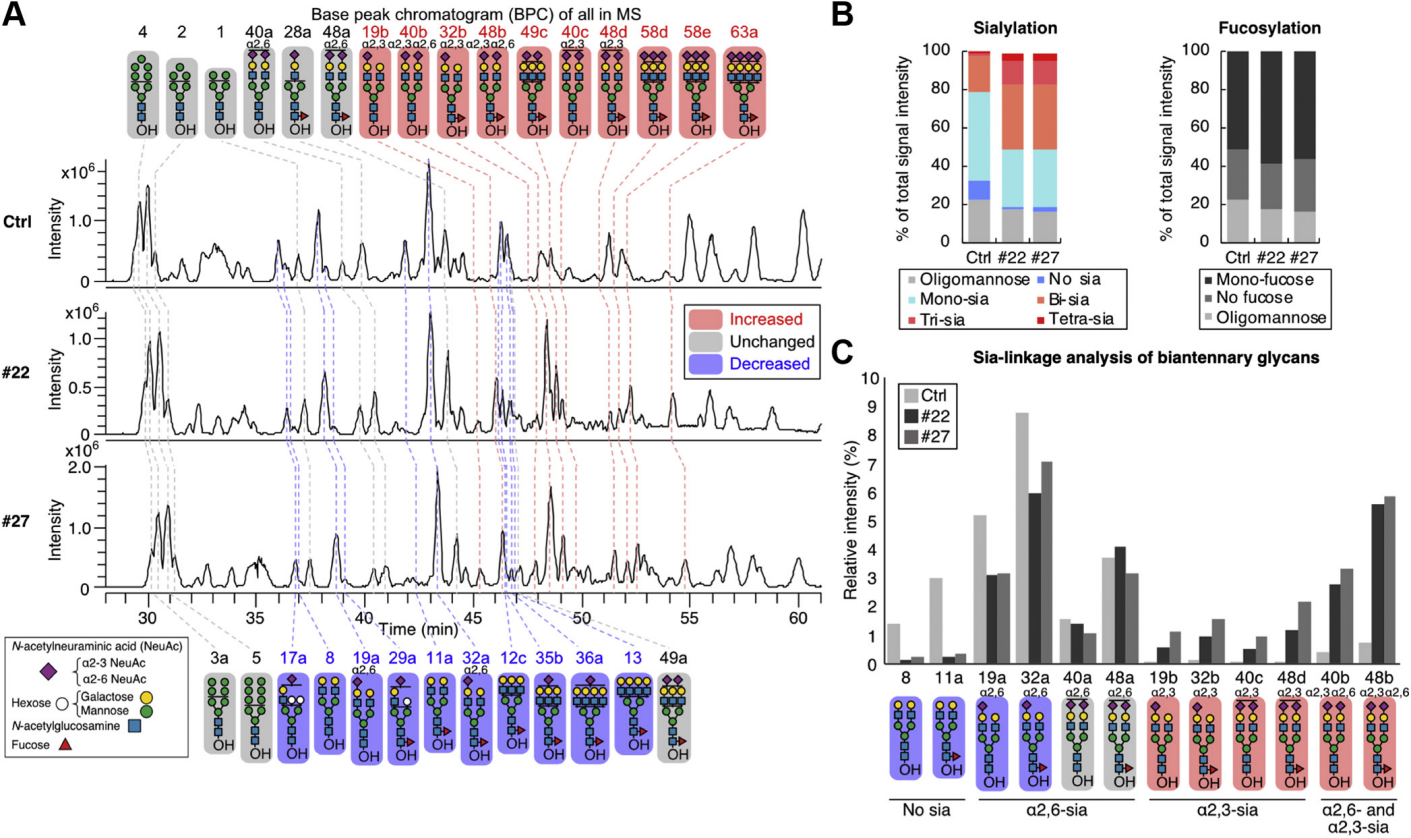
**MS analysis of glycans in control and DKD cells.***A*, base peak chromatogram (BPC) from the LC-ESI MS analysis of *N*-glycan alditols from control and DKD cells are shown. The deduced structures of the major *N*-glycans are shown. *Pink*, glycans increased in DKD; *blue*, glycans decreased in DKD; *gray*, glycans unchanged between control and DKD. *B*, *Left*, sum of the signal intensities of oligomannose glycans, nonsialylated hybrid or complex glycans, and mono-, di-, tri-, and tetra-sialylated glycans from LC-MS analysis are shown. *Right*, sum of the signal intensities of oligomannose glycans, nonfucosylated hybrid or complex glycans, and mono-fucosylated glycans from LC-MS analysis are shown. Multifucosylated glycans were not detected. *C*, LC-MS signal intensities of the major biantennary sialylated *N*-glycans derived from control and DKD cells.

To obtain more convincing evidence for the enhanced sialylation in DKD cells, we next analyzed glycan structures by MS. *N*-glycans were released from control or Rab11 DKD cells by a PNGase F treatment and reduced, and the *N*-glycan alditols were then analyzed by LC-ESI-MS (Fig. 3*A*, Figs. S1 and S2). We detected 202 glycans (Table S1) and found increases in the levels of di-, tri-, and tetra-sialylated glycans and decreases in the levels of mono- and nonsialylated *N*-glycans in DKD cells compared with control cells (Fig. 3*B*, left), clearly showing that sialylation is enhanced in DKD cells, consistent with the above results. In contrast, no remarkable differences in the relative amounts of fucosylated glycans were observed (Fig. 3*B*, right), suggesting that the effects of Rab11 knockdown on glycosylation pathways are not global but, rather, are somewhat selective to sialylation. We further carried out a linkage-specific analysis of sialic acids for the major biantennary *N*-glycans. Based on the chromatograms of *N*-glycans from a control fetuin sample whose Sia linkages have already been determined (37) (Fig. S1, left), we were able to successfully assign the Sia linkages of each glycan isomer, even though an extracted ion chromatogram (EIC) included more than one glycan with different Sia linkages (Fig. S1). As a result, we found that the amounts of α2,3- rather than α2,6-sialylated glycans were strikingly elevated in the DKD samples (Fig. 3*C*, indicated in pink). These findings show that α2,3-sialylation of *N*-glycans is especially enhanced in DKD cells.

### A candidate sialyltransferase for enhanced α2,3-sialylation in DKD cells

**Figure 4:**
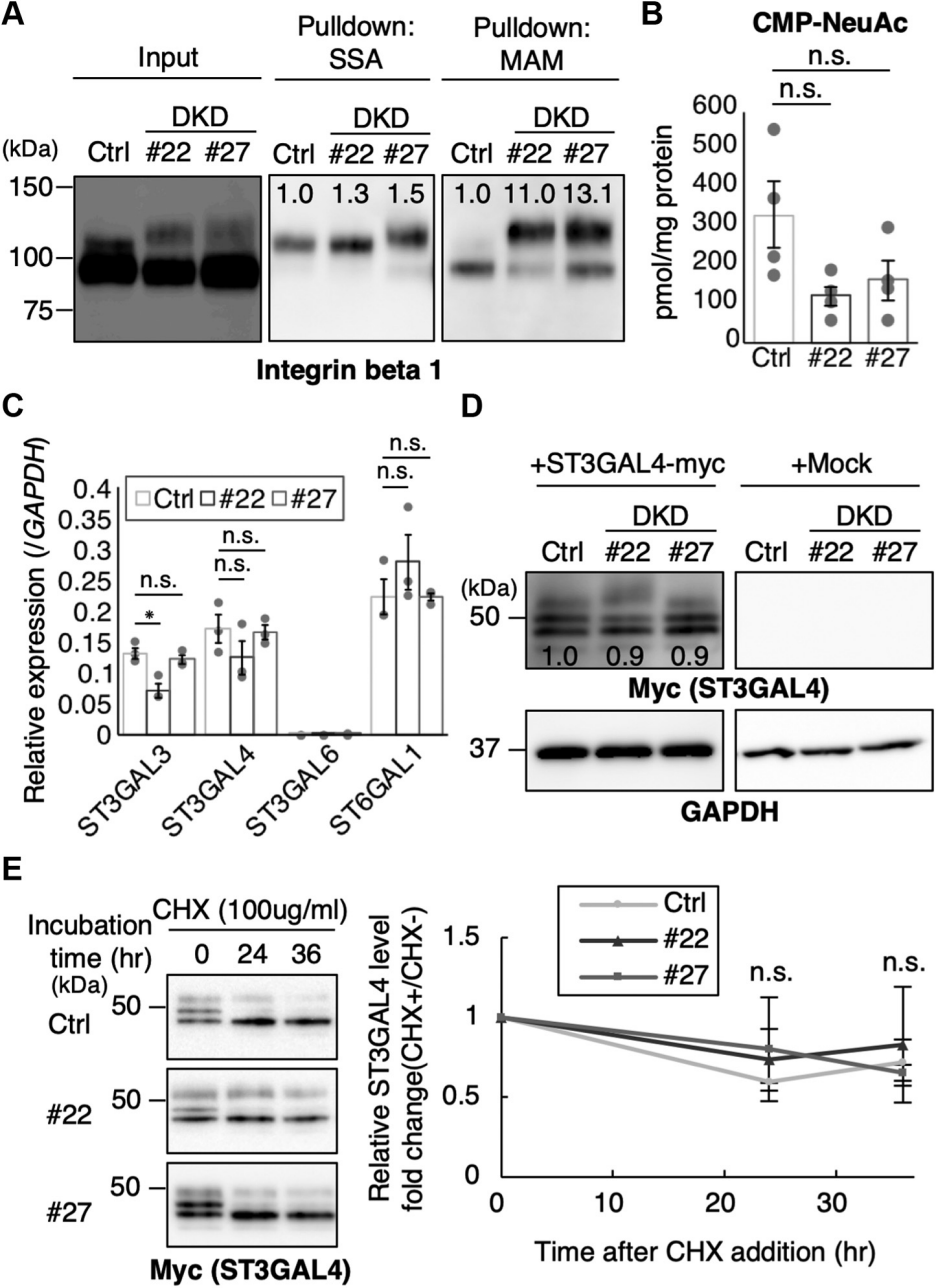
**The levels of mRNA, protein, and donor substrate of ST3GAL4 in control and DKD cells.***A*, proteins from control and DKD cells were subjected to pull-down assays with lectin agarose (SSA or MAM) and analyzed by western blotting with anti-integrin beta 1 antibody. Signal intensity of the upper band, relative to that of control sample, in the middle and right panels is displayed. *B*, the levels of CMP-Neu5Ac in control and DKD cells. *C*, the mRNA levels from control and DKD cells were quantified by real-time PCR with the *ST3GAL3*, *ST3GAL4*, *ST6GAL1*, and *GAPDH* primers. *D*, proteins from control or DKD cells transfected with the empty vector (mock) or the plasmid for ST3GAL4-myc were analyzed by western blotting with anti-myc or anti-GAPDH antibody. Signal intensity of the band in anti-myc panel relative to that of control sample is displayed. *E*, control and DKD cells were transfected with the plasmid for ST3GAL4-myc. Cycloheximide (CHX) or vehicle was added to the cells at 48 h posttransfection, and the cells were chased for 0, 24, and 36 h. Proteins in the cell lysates were blotted with anti-myc antibody. Band intensity was quantified and normalized with the vehicle controls. All the graphs in Figure 4 are shown as means ± SEM (*A* and *C–E*: *n* = 3, B: *n* = 4). Statistical analysis was performed by the Tukey–Kramer test (n.s., not significant).

To ensure the MS results showing the enhanced α2,3-sialylation of *N*-glycans in DKD cells, we performed pull-down assays with SSA (α2,6Sia) and MAM (α2,3Sia) lectins. Lysates from control and DKD HeLa cells were incubated with SSA- or MAM-agarose, and the bound integrin beta 1 was analyzed by western blotting. The reactivity of integrin beta 1 from DKD cells with MAM was drastically increased compared with control cells (Fig. 4*A*, right panel), whereas that with SSA was barely changed (Fig. 4*A*, middle panel), confirming that α2,3- but not α2,6-sialylation is enhanced in DKD cells. We also measured the amounts of CMP-*N*-acetylneuraminic acid (NeuAc), the donor substrate for sialylation, in cells and found that the level of CMP-NeuAc was decreased, rather than being increased in DKD cells compared with control cells (Fig. 4*B*), excluding the possibility that the enhanced sialylation in DKD cells was caused by an increase in the donor substrate. Combined with the fact that enhanced sialylation in DKD cells is linkage-specific but not global, we reasoned that a glycosyltransferase responsible for the α2,3-sialylation of *N*-glycans is activated or upregulated by the depletion of Rab11. A recent knockout study clearly showed that among several α2,3-siayltransferases, ST3GAL4 is the dominant enzyme for the α2,3-sialylation of integrin beta 1 *N*-glycans in HeLa cells (38). This prompted us to hypothesize that the expression or localization of ST3GAL4 is altered in DKD cells.

To address this issue, using real-time PCR, we measured the levels of *ST3GAL4* mRNA as well as other sialyltransferases that are involved in *N*-glycan biosynthesis and found that the expression of *ST3GAL4* mRNA was not upregulated in DKD cells compared with control cells (Fig. 4*C*). This excludes the possibility that the enhanced α2,3-sialylation in DKD cells was caused by the upregulation of the expression of the *ST3GAL4* gene. We next examined the level and half-life of the ST3GAL4 protein, since an aberrantly high stability of the ST3GAL4 protein might cause an enhanced α2,3-sialylation. Since the endogenous ST3GAL4 protein was difficult to detect due to its low expression level, we overexpressed myc-tagged ST3GAL4 in control and DKD HeLa cells. We first confirmed that the enhanced α2,3-sialylation was observed in ST3GAL4-myc-expressing DKD cells (Fig. S3). The steady-state levels of the expressed ST3GAL4 protein were comparable between the control and DKD cells (Fig. 4*D*). As the levels of synthesis of exogenously expressed ST3GAL4 are considered to be similar between control and DKD cells, it suggests that the stability of the ST3GAL4 protein is also unlikely altered. However, since Rab11 regulates post-Golgi trafficking, Rab11 might be involved in degradation of its target proteins. To confirm that degradation of ST3GAL4 is not affected by Rab11 knockdown, we performed cycloheximide (CHX) chase experiments. CHX is a protein synthesis inhibitor, and chasing a target protein in the presence of CHX for a certain time enables us to estimate its degradation speed. The results indicate that the degradation rates of the expressed ST3GAL4 protein in DKD cells were similar to that in control cells (Fig. 4*E*). These data show that the mRNA and protein levels of ST3GAL4 were not affected by Rab11 knockdown.

### Impaired transport of ST3GAL4 in DKD cells

**Figure 5:**
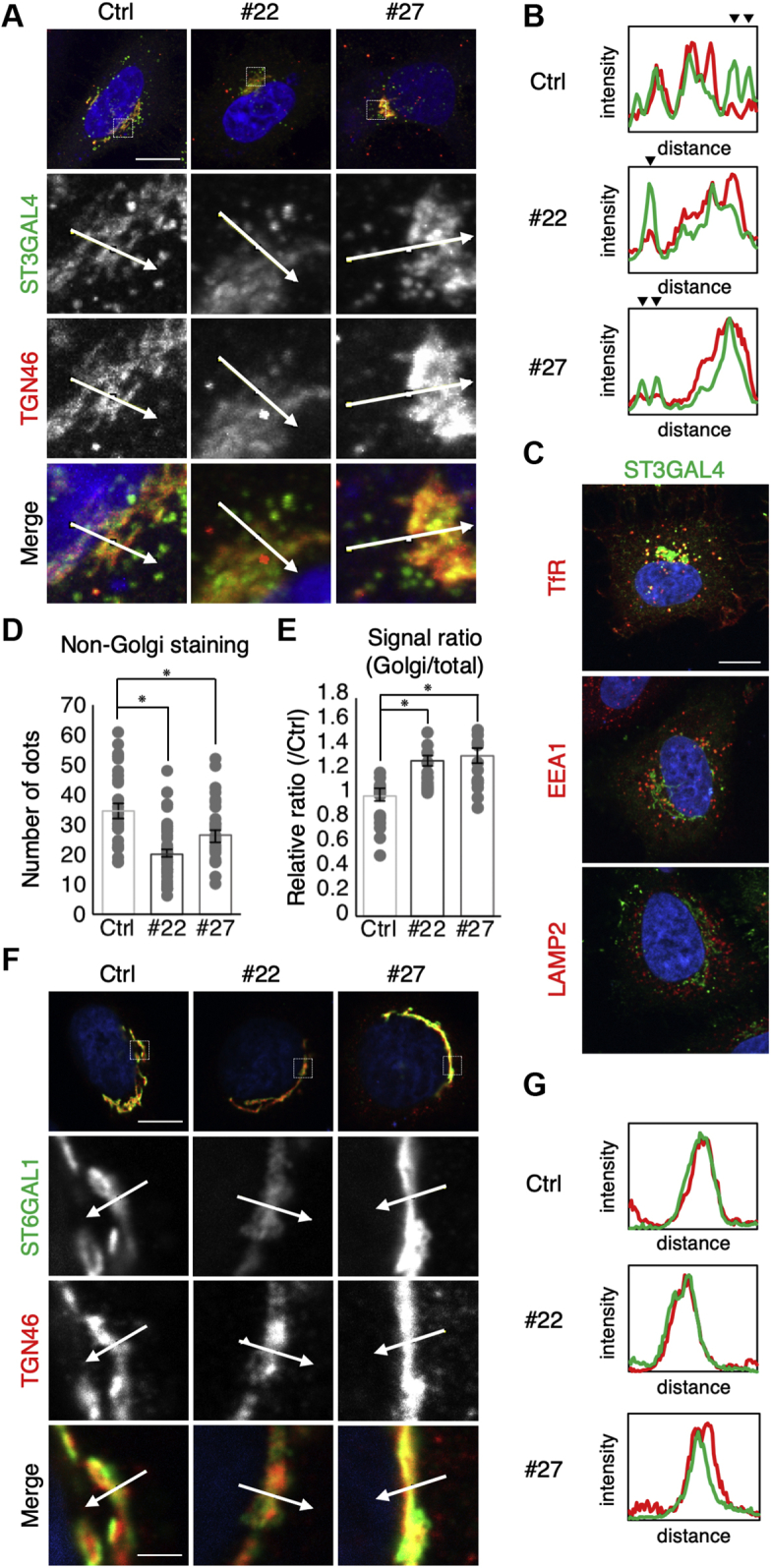
**Subcellular localization of ST3GAL4 and ST6GAL1 in control and DKD cells.***A*, *F*, cells were transfected with the plasmid encoding ST3GAL4 (*A*) or ST6GAL1 (*F*) and were immunostained for myc (ST3GAL4, *A*) or Flag (ST6GAL1, *F*) and TGN46 at 48 h posttransfection (*green*: myc or Flag, *red*: TGN46, *blue*: hoechst33342). Bars: (main images) 10 μm; (enlarged images) 2 μm. *B*, *G*, the fluorescence intensity profiles of TGN46 and either ST3GAL4 (*B*) or ST6GAL1 (G) were measured at the position marked by the arrows in A and E. The arrowheads in the graphs indicate the intensity peak of ST3GAL4 at PGCs. (C) Co-immunostaining of ST3GAL4-myc with the Transferrin receptor (TfR), EEA1 or LAMP2 in control cells (*green*: myc, *red*: TfR, EEA1 or LAMP2, *blue*: hoechst33342). Bars: 10 μm. *D*, quantification of the number of TGN46-negative ST3GAL4-positive dots in control and DKD cells. *E*, the ratios of signal intensity of Golgi-localized ST3GAL4 to that of total ST3GAL4 are shown as a value relative to that of control sample. All data are shown as means ± SEM (*D*: *n* ≧ 30, *E*: n ≧ 13). Statistical analysis was performed by Tukey–Kramer test (∗*p* < 0.05).

We next examined the subcellular localization of ST3GAL4. Since Rab11 was shown to be required for membrane transport from the TGN to recycling endosomes (39, 40), we hypothesized that the Rab11-mediated post-Golgi trafficking of ST3GAL4 could be impaired in DKD cells. Immunofluorescence staining revealed that although ST3GAL4 was mainly colocalized with TGN46 (a marker for the trans Golgi network (TGN)) (Fig. 5*A*), it was also distributed in non-Golgi vesicles (Fig. 5*B*, arrowheads). Costaining of ST3GAL4 with markers for PGCs, such as Transferrin receptor (TfR) (recycling endosomes), EEA1 (early endosomes), and LAMP2 (late endosomes and lysosomes), showed that ST3GAL4 is partially localized in PGCs and particularly in recycling endosomes (Fig. 5*C*). Furthermore, the number of ST3GAL4-positive non-Golgi dots (Fig. 5*A*) was significantly decreased in DKD cells compared with control cells (Fig. 5*D*), while the level of ST3GAL4 in the Golgi was significantly increased in DKD cells (Fig. 5*E*), suggesting that ST3GAL4 had accumulated in the TGN in DKD cells. On the other hand, the α2,6-siayltransferase ST6GAL1 was exclusively colocalized with TGN46 in control cells (Fig. 5, *F* and *G*), and the distribution was not altered in DKD cells. The change in the localization of ST3GAL4, but not ST6GAL1, is consistent with the dramatic increases in α2,3- but not α2,6-sialylated glycans in our MS analysis. Collectively, these data suggest that ST3GAL4 is transported to PGCs in a Rab11-dependent manner and that the blockade of the post-Golgi trafficking of ST3GAL4 by Rab11 knockdown results in the accumulation of ST3GAL4 in the Golgi, leading to the observed increases in the levels of α2,3-sialylated *N*-glycans produced in the Golgi.

## Discussion

**Figure 6:**
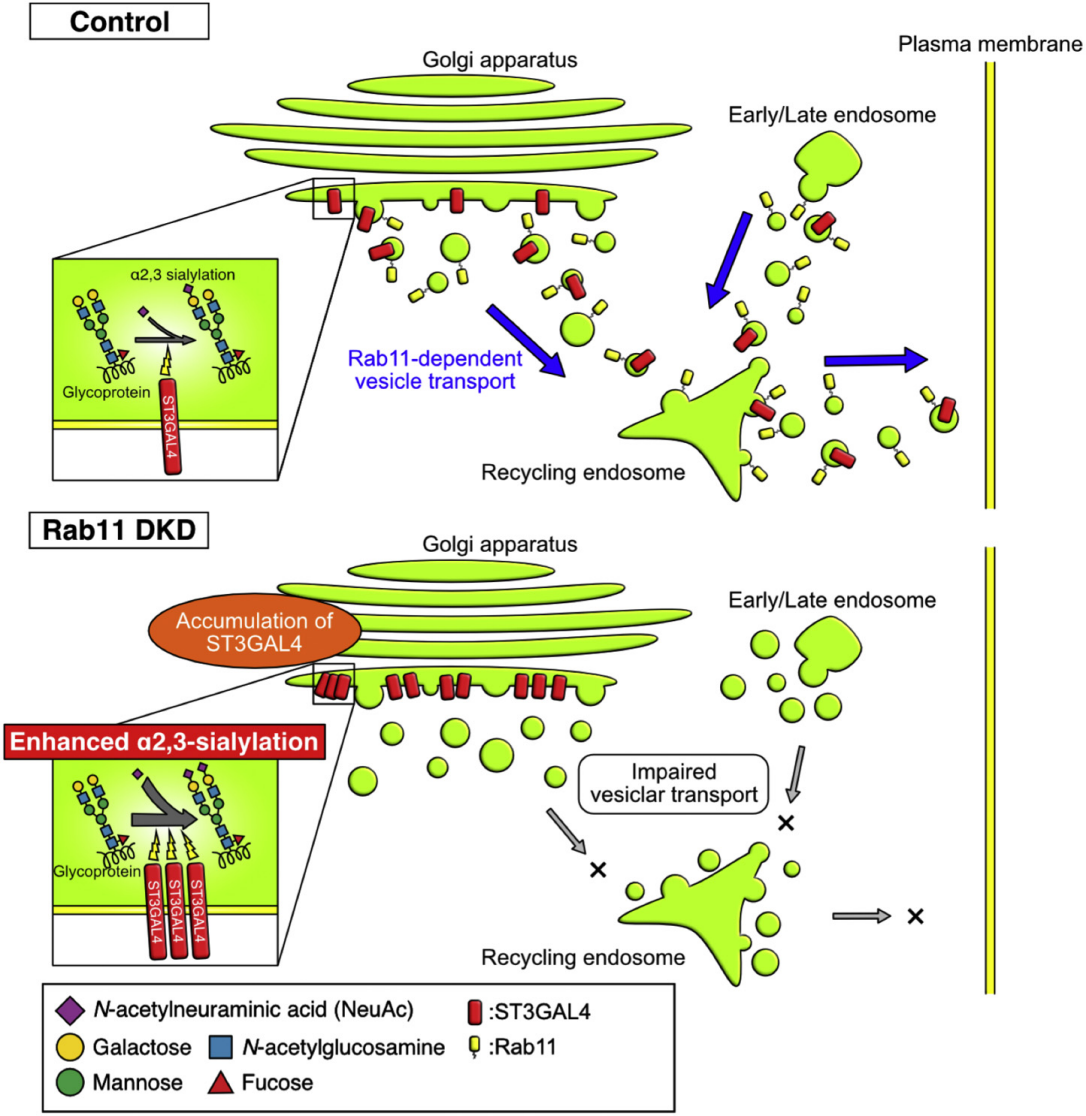
**Schematic model of this study.** Impaired endosomal transport by the knockdown of Rab11a and Rab11b results in the accumulation of ST3GAL4 in the trans-Golgi network, leading to an enhancement in the degree of α2,3-sialylation.

The findings reported in this study show that Rab11, a main regulator of membrane trafficking *via* recycling endosomes, negatively regulates α2,3-sialylation by transporting ST3GAL4 from the Golgi apparatus to PGCs (Fig. 6). Since there have been a few reports showing that molecules that participate in the post-Golgi trafficking regulate glycosylation, our findings provide new insights into how glycosylation in cells is controlled by the dynamic transport of glycosyltransferases.

Although glycosyltransferases mainly reside in the ER and the Golgi (11), some glycosyltransferases have also been reported to be also localized in PGCs. At the PM, ST8SIA1 was suggested to be involved in the synthesis of GD3 from GM3 on the membrane of neighboring cells (41). Beta-1,4-N-acetylgalactosaminyltransferase 2 (B4GALNT2) was also reported to be localized in the Golgi and the PM and exerted dual functions depending on its localization (42). Moreover, fucosyltransferase 8 (FUT8), an α1,6-fucosyltransferase that is involved in the biosynthesis of core fucose, was recently found to be partially localized in the PM (43). However, it is unclear how these enzymes are transported to the PM. Considering the fact that Rab11 is critically involved in the exocytic and endocytic recycling pathways to the PM, not only ST3GAL4 but other glycosyltransferases as well could be transported by Rab11. To date, those glycosyltransferases have been found to be involved in the pathogenesis of various diseases, including cancer growth and metastasis (44, 45). Thus, clarifying the trafficking mechanism of those enzymes and other cancer-related glycosyltransferases could lead to the development of novel strategies for suppressing the progression of cancer.

Several physiological and pathological roles of ST3GAL4 have been also reported. ST3GAL4 is involved in the biosynthesis of sialyl-Lewis^X^ (sLe^X^) (46), which is a terminal tetrasaccharide epitope composed of Siaα2-3Galβ1-4(Fucα1-3)GlcNAc-structure and serves as a ligand for selectins (47). The interaction between sLe^X^ and selectins is required for the recruitment of immune cells, and the blockade of sLe^X^ functions impairs lymphocyte homing (46), indicating that the regulation of sLe^X^ expression by ST3GAL4 can have a great impact on immunity. Moreover, *St3gal4*-deficient mice display decreased levels of von Willebrand factor (vWF) and platelets in plasma, and blood coagulation of the mutant mice is impaired (48). This abnormality is caused by faster clearance of vWF and platelets by the asialoglycoprotein receptor-1 (49), revealing that α2,3-sialylation by ST3GAL4 is essential for the clearance of blood glycoproteins and hemostasis. In addition, the upregulation of ST3GAL4 was also reported to activate the c-Met signaling pathway and accelerate tumor cell invasion (50). These findings indicate that ST3GAL4 plays key roles in immunity, hemostasis, and cancer malignancy. Our present data could lead to development of a new Rab11-targeted strategy for modulating ST3GAL4 functions in these physiological and pathological processes.

Immunofluorescence analyses showed that ST3GAL4 but not ST6GAL1 is sorted by Rab11-dependent vesicular transport (Fig. 5). This suggests that Rab11 selectively carries ST3GAL4 from the TGN. Although the mechanism for the selective transport of ST3GAL4 has not been uncovered, one possibility is that Rab11 and/or its effectors might recognize the N-terminal cytosolic region of specific glycosyltransferases, including ST3GAL4. Several reports have already shown that the N-terminal cytosolic domain of glycosyltransferases is recognized by molecules for vesicular trafficking. For example, basic amino acids in the cytosolic tails are recognized by Sar1 and are crucial for the Golgi residency of various glycosyltransferases, such as glucuronyltransferase P (GlcAT-P, also designated as B3GAT1) (51) and enzymes for glycolipid synthesis (52). Furthermore, there are two known isoforms of beta-1,4-galactosyltransferase 1 (B4GALT1), which differ in the length of the cytosolic region, and the short form is localized at the Golgi while the long form is localized in the PM (53). These findings indicate the importance of the cytosolic region of glycosyltransferases for their trafficking. To test the hypothesis that the cytosolic region of ST3GAL4, but not that of ST6GAL1, is a target of Rab11/effector, we constructed the plasmid for chimeric ST3GAL4, in which only the N-terminal cytosolic region was replaced by the ST6GAL1 counterpart. The number of non-Golgi dots of chimeric ST3GAL4 was reduced compared with wild-type (Fig. S4), suggesting that the chimeric protein was accumulated at the Golgi and that the N-terminal region of ST3GAL4 is involved in its post-Golgi trafficking. In addition, a reduction in the number of non-Golgi dots by Rab11 knockdown was smaller for the chimeric protein than wild-type. These results indicate that Rab11/effector regulates PGC transport of ST3GAL4 both directly by recognizing the cytosolic region of ST3GAL4 and indirectly by binding to another target. Our next important question will be to clarify the issue of whether Rab11/effector recognizes other glycosyltransferases for the post Golgi trafficking.

Rab11 transports various cargo molecules through multiple pathways depending on its adaptor, motor, and other effector proteins. For example, the Rab11-FIP2-Myosin Vb complex transports cargos along with actin filaments (54), while the Rab11-protrudin-Kinesin family 5a transports cargos along with microtubules (55). This indicates that associations between Rab11 and various partner molecules confer the multiple functions of this molecule. Our study, for the first time, showed that Rab11 is also involved in the transport of a glycosyltransferase, and the next important issue is to determine what Rab11 partner is required for the PGC transport of ST3GAL4. Furthermore, how this unique transport system of glycosyltransferases is involved in cellular functions and diseases is also an intriguing question to be solved in the future. Rab11 was indeed reported to be related to some diseases such as Alzheimer’s disease and Huntington’s disease (56, 57), but the detailed mechanisms for how Rab11 is involved in these diseases remain poorly understood. A phenotype or pathology caused by a Rab11 dysfunction could be partially attributed to the abnormal transport of glycosyltransferases and resultant aberrant glycosylation. Future studies focusing on the Rab-mediated post-Golgi trafficking of glycosyltransferases promise to unveil a novel regulation mechanism of glycosylation, leading to elucidation of a new link between Rab-mediated membrane transport and glycan function.

## Experimental procedures

### Antibodies and lectins

The following antibodies and reagents were used: anti-LAMP1 (ab24170, abcam), anti-LAMP2 (clone H4B4, Developmental Studies Hybridoma Bank), anti-Lamin B (sc-6216, Santa Cruz), anti-Rab11 (clone 47, BD Biosciences), anti-GAPDH (6C5, EMD Millipore), anti-DYKDDDDK (clone L5, Novus Biologicals), anti-myc (4A6, Merck Millipore) (562, MBL Life Science), anti-TGN46 (AHP500GT, Bio-Rad), anti-integrin beta 1 (ab183666, abcam), anti-EEA1 (610456, BD Biosciences), anti-Transferrin receptor (3C11F11, proteintech), anti-mouse IgG HRP linked whole antibody from sheep (NA931V, GE Healthcare), anti-rabbit IgG HRP linked whole antibody from donkey (NA934V, GE Healthcare), anti-rat IgG HRP linked whole antibody from goat (NA935, GE Healthcare), Hoechst 33342 (Dojindo), donkey anti-mouse IgG (H+L) highly cross-adsorbed secondary antibody, Alexa Fluor 594 (A-21203, Invitrogen), donkey anti-rabbit IgG (H+L) highly cross-adsorbed secondary antibody, Alexa Fluor 488 (A-11008, Invitrogen), donkey anti-rat IgG (H+L) highly cross-adsorbed secondary antibody, Alexa Fluor 488 (A-21208, Invitrogen), Donkey anti-Sheep IgG (H+L) Cross-Adsorbed Secondary Antibody, Alexa Fluor 594 (A-11016, Invitrogen). Biotinylated PhoSL was kindly provided by J Chemical (58), and other biotinylated lectins were purchased from J Chemical (ConA, #J203; L4-PHA, #J212; SSA, #J218; MAM, #J210).

### Plasmid

A pcDNA-Flag vector encoding rat ST6GAL1 was provided by Dr Shinobu Kitazume (Fukushima Medical University) (59). A pcDNA6/myc-His A vector encoding human ST3GAL4 was constructed in a previous report (60). A pcDNA6/myc-His A vector encoding chimeric human ST3GAL4 having cytosolic region of ST6GAL1 was constructed by using a pcDNA-Flag vector encoding rat ST6GAL1, a pcDNA6/myc-His A vector encoding human ST3GAL4 and iVEC3 (National BioResource Project (NIG): *E. coli*) (61). Briefly, we first amplified the DNA fragment consisting of the ST3GAL4 sequence (encoding the transmembrane and C-terminal domains) and pcDNA6/myc-His A vector sequence by PCR with PrimeSTAR Max DNA Polymerase (TAKARA) using the pcDNA6/myc-His A/human ST3GAL4 as a template. The following primers were used: CTCCTGGCCATGTTGGCTCT (forward), GAATTCCACCACACTGGACT (reverse). We designed and annealed the complementary oligonucleotides, which encode the N-terminal domain of ST6GAL1 with 15-bp overlapping ends homologous to the above PCR product. The following oligonucleotides were used: AGTGTGGTGGAATTCATGATTCACACCAACCTGAAGAAAAAGCTCCTGGCCATGTTG (forward), CAACATGGCCAGGAGCTTTTTCTTCAGGTTGGTGTGAATCATGAATTCCACCACACT (reverse). The PCR product and the annealed nucleotides were mixed and added to iVEC3.

### Cell culture and transfection

HeLa cells expressing Rab11a and Rab11b shRNAs in which Rab11a and Rab11b are stably knocked down (DKD cells) were established as described in a previous report (28). DKD cells were cultured in Dulbecco's modified eagle medium containing 10% fetal bovine serum, 2 μg/ml puromycin, and 400 μg/ml G418. TransIT-LT1 (Takara Bio) was used for plasmid transfection according to the manufacturer’s instruction. Briefly, TransIT-LT1 and the plasmid solution were mixed at a ratio of 3:1 in Opti-MEM. After a 20-min incubation at room temperature, the mixture was added to the culture medium, followed by incubation at 37 °C for 48 h.

### Western and lectin blotting

Cells were collected in PBS and lysed with PBS containing 0.2% Triton X-100 and cOmplete Mini EDTA-free protease inhibitor cocktail (Roche). The concentration of protein in the cell lysate was measured using Protein Assay Reagent (BIO-RAD, #500-0113, 0114, 0115) according to the manufacturer’s instruction. The cell lysate was then boiled at 95 °C for 5 min in SDS sample buffer composed of 50 mM Tris pH 6.8, 2% SDS, 2.5% 2-mercaptoethanol, 9% glycerol, and 1% Bromophenol blue. The proteins (western blotting, 20 μg/well; lectin blotting with MAM, 20 μg/well; lectin blotting with ConA, PhoSL, L4-PHA, SSA or RCA120, 5 μg/well) were separated by SDS-PAGE and then transferred to poly(vinylidene fluoride) (PVDF) or nitrocellulose membranes. The membranes were blocked with 5% skim milk (Nacalai tesque) in TBS containing 0.1% Tween 20 tris-buffered saline (TBS-T) (or blocked with TBS-T for lectin blot). The membranes were then incubated with primary antibodies or biotinylated lectin, followed by HRP-conjugated secondary antibodies or HRP-conjugated streptavidin (VECTASTAIN ABC Standard Kit). Antibodies were diluted with 5% skim milk in TBS-T, and lectins and streptavidin were diluted with TBS-T. Proteins were detected with ECL Prime Western Blotting Detection Reagents (GE Healthcare) using an ImageQuant LAS-4000mini (GE Healthcare).

### Glycosidase treatment of cell lysate

Twenty microgram of proteins that had been prepared and quantified by the same methods as those in “Western and lectin blotting” section was denatured by boiling in SDS sample buffer. The samples were diluted to 20 μl with SDS sample buffer, and 1 μl of 20% Triton X-100 and 1 μl of 25 x cOmplete Mini EDTA-free protease inhibitor cocktails were added to the mixture. The mixtures were incubated with 500 units of peptide *N*-glycosidase F (PNGase F) (New England BioLabs) or Endoglycosidase H (Endo H) (New England BioLabs) at 37 °C. For neuraminidase treatment, 20 μg of proteins was incubated with 0.01 U of Neuraminidase (from *Arthrobacter ureafaciens*) in 100 mM ammonium acetate (pH 5.0) at 37 °C. After 0-, 1-, 3-h incubation, the samples were boiled in SDS sample buffer and subjected to western blotting.

### Neuraminidase treatment of PVDF membrane

Protein-transferred membranes were washed with TBS-T twice and treated with 0.1 U of Neuraminidase in 50 mM sodium acetate for 3 h at 37 °C. The membrane was then washed three times with TBS-T and used for western blotting.

### Click chemistry

Cells were incubated with 50 μM Click-IT ManNAz (ThermoFisher Scientific) for 2 days. Cells were collected in PBS and lysed with PBS containing 0.2% Triton X-100 and cOmplete Mini EDTA-free protease inhibitor cocktail.

The lysates were reacted with 10 μM biotin alkyne (Thermo Fisher Scientific) using Click-iT Protein Reaction Buffer Kit (ThermoFisher Scientific) according to the manufacturer’s instruction. The samples were then analyzed by SDS-PAGE and blotting with HRP-conjugated streptavidin.

### Analysis of extracellular proteins

Culture media were replaced with 10 ml (for a 10-cm dish) of Opti-MEM I Reduced Serum Medium (31985070, Gibco) without adding any serum or supplement, followed by a 72-h incubation. The media were collected and centrifuged at 200*g* for 5 min to remove cell debris. All the supernatants were mixed with 333 μl of 5 M NaCl and 26 ml of ethanol and subsequently incubated at –20 °C for 30 min to precipitate the secreted proteins. The mixtures were centrifuged at 12,000*g* for 30 min, and the pellets were washed with 1 ml of 70% ethanol and centrifuged again at 12,000*g* for 15 min. The precipitated proteins were dissolved by sonication and boiled in 200 μl of SDS sample buffer. The samples were subjected to SDS-PAGE, and total proteins were stained with GelCode Blue Stain Reagent (24590, Thermo Scientific) according to the manufacturer’s instruction.

### Glycan analysis of cell membrane proteins by LC-ESI MS

*N*-Glycans from cell membrane proteins were released, reduced to alditol *N*-glycans, and analyzed by LC-ESI MS, according to previous procedures (37, 62, 63) with modifications as follows. HeLa cells (2 × 10^7^ cells) were homogenized in 2 ml of Lysis-buffer (50 mM Tris-HCl, pH 7.4, 0.1 M NaCl, 1 mM EDTA and protease inhibitor cocktail (Roche)) using a polytron homogenizer (seven times for 15 s on ice bath), followed by centrifugation at 2000*g* for 20 min at 4 °C to remove nuclei and unbroken cells. The supernatant was diluted with 2 ml of Tris-buffer (50 mM Tris-HCl, pH 7.4, 0.1 M NaCl) and then ultracentrifuged at 120,000*g* for 80 min at 4 °C. The membrane pellet was suspended in 100 μl of the Tris-buffer, followed by the addition of 400 μl of the Tris-buffer containing 1% Triton X-114 with pipetting. The lysate was incubated on ice for 10 min and then at 37 °C for 20 min, followed by phase partitioning by centrifugation at 1940*g* for 2 min. The upper aqueous phase was removed, and the lower detergent phase was mixed with 1 ml of ice-cold acetone and stored at –25 °C overnight. After centrifugation for 2 min at 1940*g*, the precipitated membrane proteins were dissolved in 10 μl of 8 M urea and spotted (2.5 μl × four times) onto an ethanol-pretreated PVDF membrane. After drying at room temperature for 4 h, the membrane was washed once for 1 min with ethanol and then three times for 1 min with water. The protein on the membrane was stained for 5 min with Direct Blue 71 (Sigma Aldrich) (800 μl solution A [0.1% Direct Blue 71] in 10 ml solution B [acetic acid:ethanol:water = 1:4:5]). After destaining with solution B for 1 min, the membrane was dried at 3 h room temperature. The protein spots were excised from the PVDF membrane and placed into a well of a 96-well plate. The spots were covered with 100 μl of 1% (w/v) polyvinylpyrrolidone 40,000 in 50% (v/v) methanol, agitated for 20 min, and washed with water (100 μl × five times). PNGase F (2 U in 10 μl of 20 mM phosphate buffer, pH 7.3, Roche) was added to the well and the spots were incubated at 37 °C for 15 min, followed by the addition of 10 μl of water, and incubated at 37 °C overnight. The samples were sonicated (in the 96-well plate) for 10 min and the released *N*-glycans (20 μl) were transferred to 1.5-mL polypropylene tubes. The sample well was washed with water (50 μl twice), and the washings were combined. To completely generate the reducing terminus, ammonium acetate buffer (100 mM, pH 5.0, 20 μl) was added to the released *N*-glycans and the suspension allowed to stand for 1 h at room temperature. After evaporating to dryness, the *N*-glycans were reduced with 20 μl of 1 M NaBH_4_ in 50 mM KOH at 50 °C for 3 h. One microliter of acetic acid was added to terminate the reaction, and the alditol *N*-glycans were desalted using a cation-exchange column. The alditol *N*-glycans were eluted with water (50 μl twice), dried, and the remaining borate was removed by the addition of (100 μl × three times) methanol and drying under vacuum. The alditol *N*-glycans were resuspended in 10 mM ammonium bicarbonate (15 μl) immediately before glycan analysis by liquid chromatography–electrospray ionization–mass spectrometry (LC-ESI-MS). The alditol *N*-glycans (injection volume; 8 μl) were separated on a carbon column (5 μm HyperCarb, 1 mm I.D. × 100 μm, Thermo Fisher Scientific) using an Accela HPLC pump (flow rate: 50 μl/min, column oven: 40 °C) under the following gradient conditions involving a sequence of isocratic and two segmented linear gradients: 0–8 min, 10 mM ammonium bicarbonate; 8–38 min, 6.75-15.75% (v/v) acetonitrile in 10 mM ammonium bicarbonate; 38–73 min, 15.75–40.5% (v/v) acetonitrile in 10 mM ammonium bicarbonate; increasing to 81% (v/v) acetonitrile in 10 mM ammonium bicarbonate for 7 min; and re-equilibrated with 10 mM ammonium bicarbonate for 15 min. The eluate was continuously introduced into an ESI source (LTQ Orbitrap XL, Thermo Fisher Scientific). MS spectra were obtained in the negative ion mode using Orbitrap MS (mass range m/z 500 to m/z 2500; capillary temperature: 300 °C, source voltage: 3.0 kV; capillary voltage: −18 V; tube lens voltage: −112.80 V), and MS/MS spectra were obtained using Iontrap MS by CID.

The monoisotopic mass of each alditol *N*-glycan was assigned to a possible monosaccharide composition using the GlycoMod software tool (mass tolerance for precursor ions of ±0.01 Da, https://web.expasy.org/glycomod/), and the proposed glycan structures were further verified through MS/MS fragment patterns and retention time based on a previous report (64). Xcalibur software ver. 2.2. (Thermo Fisher Scientific) was used to show the base peak chromatogram (BPC), extracted ion chromatogram (EIC) and to analyze MS and MS/MS data. The relative abundances (%) of each glycan structures were calculated by setting the total peak intensities of all detected alditol *N*-glycans in each EIC as 100%.

### Analysis of cellular nucleotide sugars

Nucleotide sugars from control and DKD cells were prepared and quantified by LC-ESI-MS/MS as reported previously (65, 66). Briefly, cells plated on a 10 cm dish were washed once with cold PBS, collected in ice-cold 70% ethanol (2 ml), and spiked with an unnatural nucleotide sugar derivative, GDP-Glc (1 nmol). The extracts were centrifuged at 16,000*g* for 15 min at 4 °C, and the supernatants were lyophilized. The freeze-dried samples were dissolved in 1 ml of 10 mM NH_4_HCO_3_ and purified on an Envi-Carb column as reported previously (66). For measuring the protein concentrations, the precipitate of 70% ethanol extraction was dissolved in 2% SDS, followed by quantification of protein concentration using Pierce BCA Protein Assay Kit (#23227).

LC-ESI-MS/MS was performed on an LCMS-8060 (Shimadzu) coupled with a Nexera HPLC system (Shimadzu). Chromatography was performed on a Zwitterionic (ZIC) column with phosphocholine phase (ZIC-cHILIC, 2.1 mm i.d. × 150 mm, 3 μm; Merck SeQuant) (65). Analysis of nucleotide sugars was conducted in the multiple reaction monitoring (MRM) mode using specific precursor–product ion pairs for CMP-NeuAc, *m/z* 613(Q1)→322 (Q3). The nucleotide sugar levels were normalized as pmol/mg proteins.

### Lectin pull-down

Lectin agaroses (SSA or MAM) were washed three times with PBS containing 0.2% Triton X-100. In total, 500 μg of lysed proteins was mixed with washed agarose and incubated overnight at 4 °C. The mixtures were then centrifuged at 1000*g* for 1 min at 4 °C, and the supernatants were removed. The beads were washed with PBS containing 0.2% Triton X-100 for three times and boiled at 95 °C for 5 min in SDS sample buffer. The supernatants of each sample were subjected to western blotting.

### Cycloheximide chase

Cycloheximide (Wako) was added to the culture medium at a concentration of 100 μg/ml. After 0-, 24-, 36-, 48-h periods of incubation, the cells were collected and lysed, and the lysates were subjected to western blotting.

### Immunofluorescence staining

Cells, seeded on glass coverslips, were washed with PBS, fixed with 4% PFA in PBS for 20 min at room temperature, and washed again with PBS. The fixed cells were permeabilized by treatment with PBS containing 0.2% Triton X-100 for 4 min at room temperature. The cells were blocked with 3% BSA in PBS for 30 min, then the cells were incubated with primary antibodies for 60 min, followed by secondary antibodies and 4',6-diamidino-2-phenylindole for 45 min. Fluorescence was visualized using a ZEN 3 LSM900 confocal microscope (ZEISS), and data acquisition and quantification of intensities were carried out using Fiji (67). To quantify the signals in the Golgi, we first converted acquired images to 8 bit and reduced background by using threshold “Moments.” Then, we created images that display only colocalized signals by using the “AND” command in image Calculator and measured integrated signal density.

### RNA extraction, reverse transcription, and real-time PCR

Total RNA from cultured cells was extracted using TRIzol Reagent (Invitrogen) according to the manufacturer’s instruction. Extracted total RNA was reverse-transcribed using Superscript IV (Invitrogen). For real-time PCR, cDNA was mixed with THUNDERBIRD SYBR qPCR Mix (TOYOBO) and amplified using Applied Biosystems 7500 Real-time PCR System. The levels of mRNA were normalized to the corresponding GAPDH levels. The following primers were used: CTGCCCAAGGAGAGCATTAG (*ST6GAL1*, forward), GCCCACATCTTGTTGGAAGT (*ST6GAL1*, reverse), CTATGACATTGTGGTGAGACTGA (*ST3GAL3*, forward), CTCTCTCCTTGTAGACGATGTATTT (*ST3GAL3*, reverse) (38), GGGAGATGCCATCAACAAGT (*ST3GAL4*, forward), GGAAGTCCATTGCCTTGAAA (*ST3GAL4*, reverse) CTATAAATTGAGCCCGCAGCC (*GAPDH*, forward), AACATGTAAACCATGTAGTTGAGGT (*GAPDH*, reverse).

## Data availability

Glycomic raw data and identification results for the glycan-structure analyses have been deposited to the GlycoPOST (announced ID: GPST000130). All the other data are contained in the article.

## Conflict of interest

The authors declare that they have no conflicts of interest with the contents of this article.
