# Supplementary material for: Rab11-mediated post-Golgi transport of the sialyltransferase ST3GAL4 suggests a new mechanism for regulating glycosylation
Source: J Biol Chem. 2021 Jan 30;296:100354. doi: 10.1016/j.jbc.2021.100354 (PMC7949161; doi:10.1016/j.jbc.2021.100354)
Supplement: Supplementary file 2 — Figures S1 to S4 [file mmc2.pdf]

Supporting Information

Rab11 depletion enhances the α2,3-sialylation of *N*-glycans and the Golgi localization of ST3GAL4

Masato Kitano, Yasuhiko Kizuka, Tomoaki Sobajima, Miyako Nakano, Kazuki Nakajima, Ryo Misaki, Saki Itoyama, Yoichiro Harada, Akihiro Harada, Eiji Miyoshi, Naoyuki Taniguchi

Correspondence:
Eiji Miyoshi, M.D., Ph.D.,
E-mail: emiyoshi@sahs.med.osaka-u.ac.jp

Naoyuki Taniguchi, M.D., Ph.D.,
Email: glycotani@mc.pref.osaka.jp

This Supplemental information includes:

Fig. S1-4 (included in this PDF)
Table S1 (separate excel file)

Supplemental Figure S1

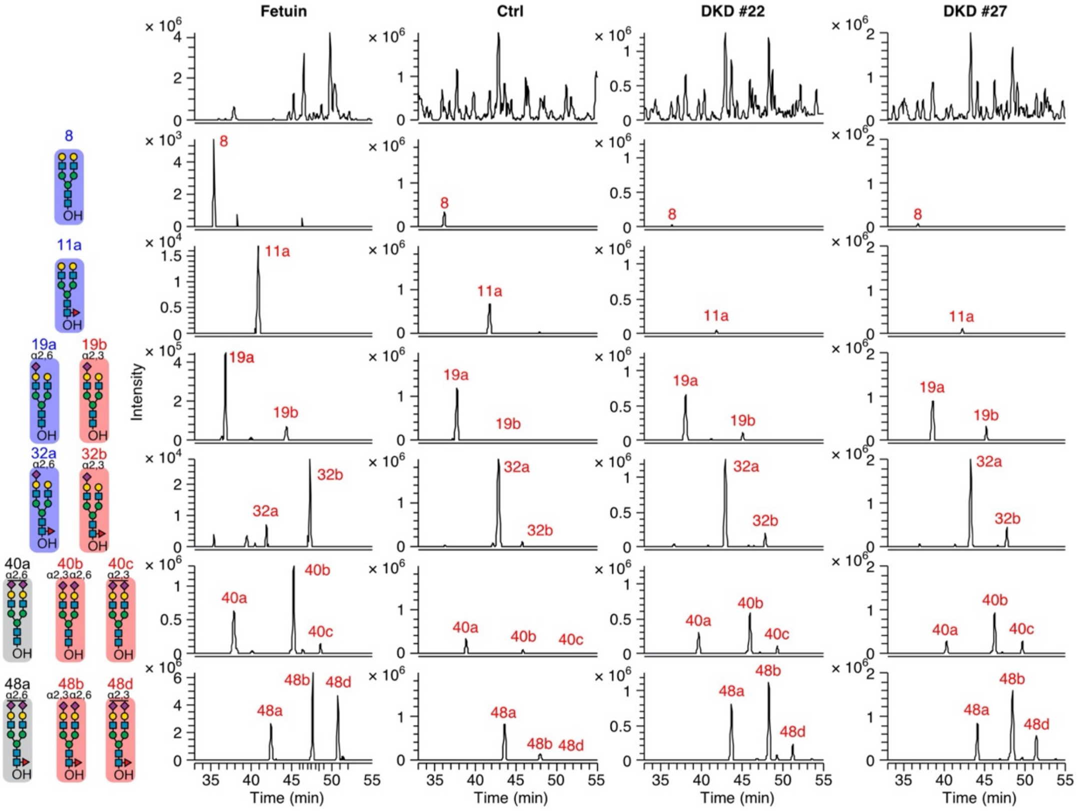

Supplemental Fig. S1. LC-MS analysis of *N*-glycans from control and DKD cells. Extracted ion chromatograms (EICs) of the major sialylated *N*-glycans shown in Fig. 3C. *N*-Glycans released from Fetuin, whose Sia linkages were already known, were analyzed as standards.

Supplemental Figure S2

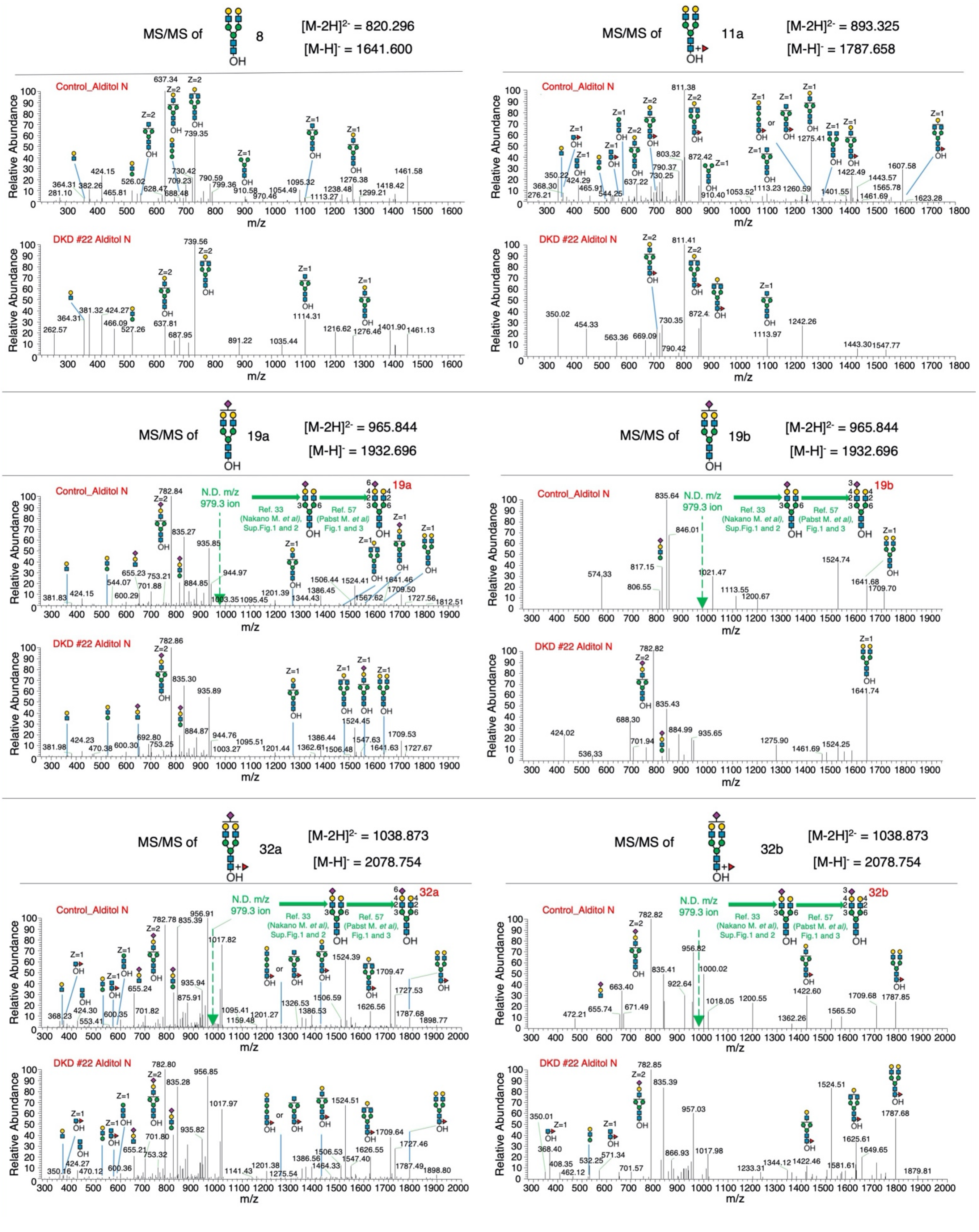

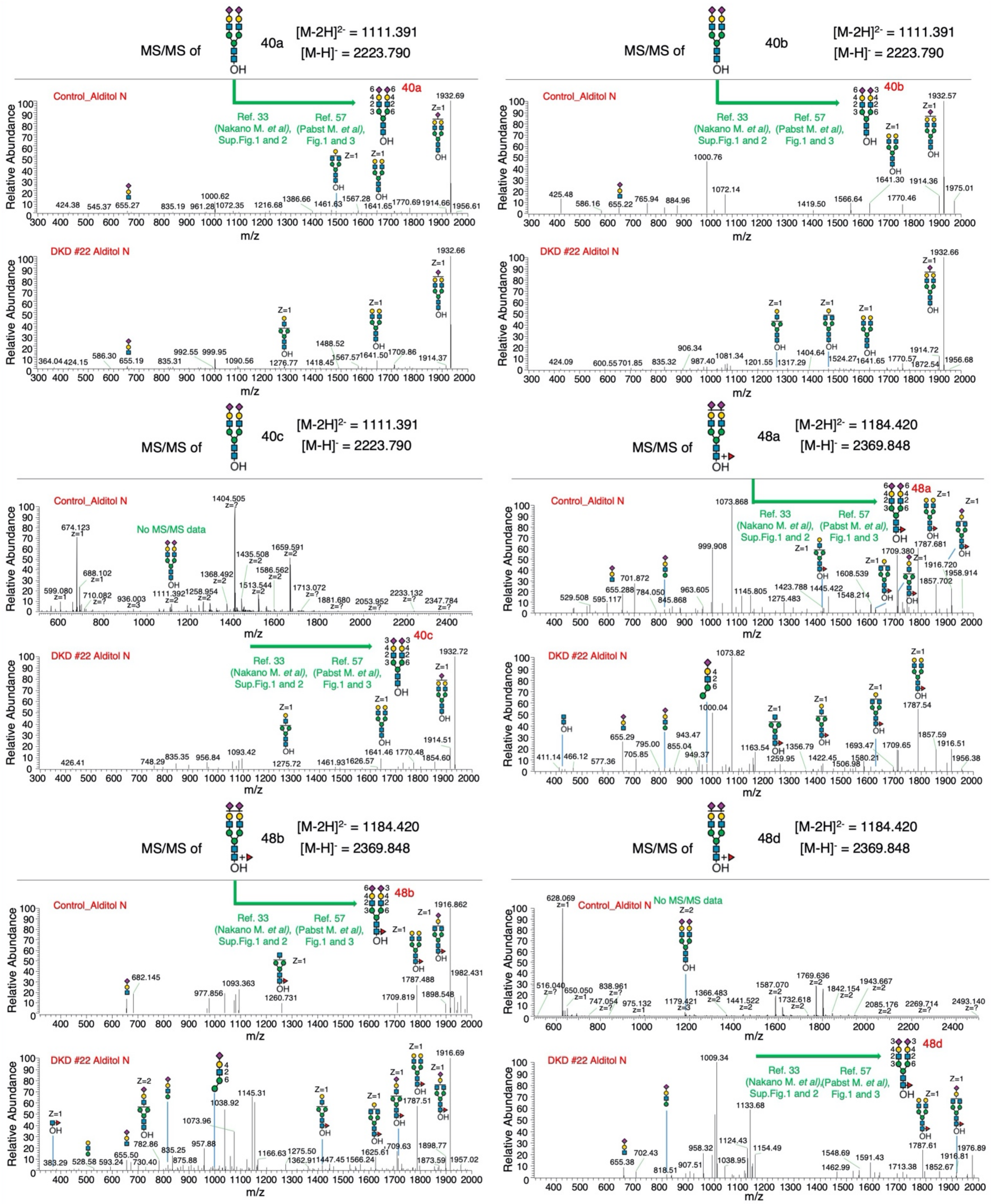

Supplemental Fig. S2. MS/MS spectra of *N*-glycans from control and DKD cells. The spectra of MS/MS analysis of the representative *N*-glycans from control and DKD cells are shown.

Supplemental Figure S3

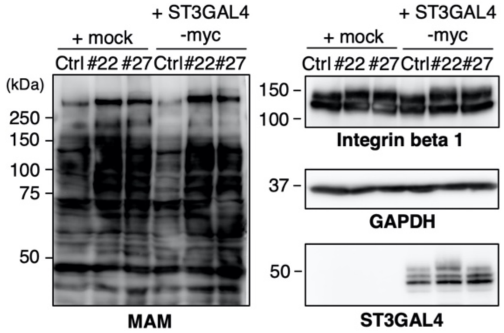

Supplemental Fig. S3 Enhanced α2,3-sialylation by Rab11 knockdown in ST3GAL4-expressing cells. Proteins from control or DKD cells transfected with the empty vector (mock) or the plasmid for ST3GAL4-myc were analyzed by lectin blotting with MAM lectin and by western blotting with anti-integrin beta 1, anti-GAPDH, or anti-myc (ST3GAL4) antibody.

Supplemental Figure S4

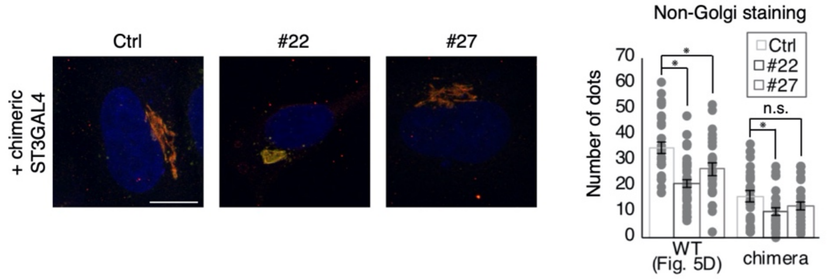

Supplemental Fig. S4. Localization analysis of chimeric ST3GAL4 having cytosolic region of ST6GAL1. (Left) Cells were transfected with the plasmid encoding ST3GAL4 chimera (composed of N-terminal cytosolic region of ST6GAL1 and transmembrane and luminal domains of ST3GAL4) and were immunostained for myc-tagged ST3GAL4 chimera and TGN46 at 48 hrs post transfection (green: myc, red: TGN46, blue: hoechst33342). Bars: 10 µm. (Right) Quantification of the number of TGN46-negative ST3GAL4-positive dots in control and DKD cells for wild-type (left, same as Fig. 5D) and ST3GAL4 chimera (right). Data are shown as means ± SEM ($n \geqq 30$). Statistical analysis was performed by Tukey-Kramer test (*, $P < 0.05$, n.s., not significant).
